# Corneal confocal microscopy identifies small fibre damage and progression of diabetic neuropathy

**DOI:** 10.1038/s41598-021-81302-8

**Published:** 2021-01-21

**Authors:** Shaishav Dhage, Maryam Ferdousi, Safwaan Adam, Jan Hoong Ho, Alise Kalteniece, Shazli Azmi, Uazman Alam, Georgios Ponirakis, Ioannis Petropoulos, Andrew J. Atkinson, Andrew Marshall, Maria Jeziorska, Handrean Soran, Rayaz A. Malik

**Affiliations:** 1 Department of Medicine, Manchester University NHS Foundation Trust, Manchester, UK; 2 Cardiovascular Research Group, University of Manchester, Manchester, UK; 3 The Christie NHS Foundation Trust, Manchester, UK; 4 Institute of Cardiovascular and Metabolic Medicine and The Pain Research Institute, University of Liverpool & Liverpool University NHS Hospital Trust, Liverpool, UK; 5 Department of Medicine, Weill Cornell Medicine-Qatar, Qatar Foundation, Education City, Doha, Qatar; 6 Institute of Life Course and Medical Sciences and The Pain Research Institute, University of Liverpool & Liverpool University NHS Hospital Trust, Liverpool, UK

**Keywords:** Diabetes, Diabetes complications

## Abstract

Accurately quantifying the progression of diabetic peripheral neuropathy is key to identify individuals who will progress to foot ulceration and to power clinical intervention trials. We have undertaken detailed neuropathy phenotyping to assess the longitudinal utility of different measures of neuropathy in patients with diabetes. Nineteen patients with diabetes (age 52.5 ± 14.7 years, duration of diabetes 26.0 ± 13.8 years) and 19 healthy controls underwent assessment of symptoms and signs of neuropathy, quantitative sensory testing, autonomic nerve function, neurophysiology, intra-epidermal nerve fibre density (IENFD) and corneal confocal microscopy (CCM) to quantify corneal nerve fibre density (CNFD), branch density (CNBD) and fibre length (CNFL). Mean follow-up was 6.5 years. Glycated haemoglobin (*p* = 0.04), low-density lipoprotein-cholesterol (LDL-C) (*p* = 0.0009) and urinary albumin creatinine ratio (*p* < 0.0001) improved. Neuropathy symptom profile (*p* = 0.03), neuropathy disability score (*p* = 0.04), vibration perception threshold (*p* = 0.02), cold perception threshold (*p* = 0.006), CNFD (*p* = 0.03), CNBD (*p* < 0.0001), CNFL (*p* < 0.0001), IENFD (*p* = 0.04), sural (*p* = 0.02) and peroneal motor nerve conduction velocity (*p* = 0.03) deteriorated significantly. Change (∆) in CNFL correlated with ∆CPT (*p* = 0.006) and ∆Expiration/Inspiration ratio (*p* = 0.002) and ∆IENFD correlated with ∆CNFD (*p* = 0.005), ∆CNBD (*p* = 0.02) and ∆CNFL (*p* = 0.01). This study shows worsening of diabetic neuropathy across a range of neuropathy measures, especially CCM, despite an improvement in HbA1c and LDL-C. It further supports the utility of CCM as a rapid, non-invasive surrogate measure of diabetic neuropathy.

## Introduction

The natural history of diabetic peripheral neuropathy (DPN) is poorly defined with limited studies assessing progression of neuropathy^1^. As a consequence, clinical trials of disease modifying therapies in patients with diabetic neuropathy have not been able to identify the optimal neuropathy end points to adequately assess progression or improvement in DPN^2^. Indeed, whilst the DCCT in patients with T1DM showed that intensive glycaemic control reduced the incidence of clinical DPN and nerve conduction abnormalities by 60%^3^; in patients with T2DM, the UKPDS^4^ and VA-CSDM trial^5^ reported no effect on DPN and cardiac autonomic neuropathy and whilst the Kumamoto study^6^ showed a prevention of nerve conduction slowing, the ACCORD trial^7^ showed no effect on VPT over 6-years.

Quantitative sensory testing (QST) is relatively easy to perform but has limited reproducibility and a high degree of subjectivity^8^. Nerve conduction studies (NCS) are the established ‘gold standard’ for evaluating DPN but require standardization in a clinical trial and cannot evaluate small fibres^9^. Whilst small nerve fibre damage and repair can be identified by performing a skin biopsy and quantifying intra-epidermal nerve fibre density (IENFD), it is invasive and requires expertise^10–12^. Other techniques for the assessment of small nerve fibres include microneurography, Laser doppler image flare (LDIflare), nociceptive-evoked potentials and electrochemical skin conductance, but have considerable variability and are not routinely available^13,14^. Corneal confocal microscopy (CCM) is a rapid non-invasive imaging technique for the quantitative assessment of small fibre damage. Several studies have shown that it has good diagnostic utility for sub-clinical DPN, predicts incident DPN^15,16^ and correlates with other measures of neuropathy^16^. Furthermore, automated quantification of corneal nerve parameters allows rapid, unbiased and objective assessment of small fibre damage^17^ with comparable diagnostic capability to IENFD^18,19^.

Longitudinal studies of patients with diabetic neuropathy have been of relatively short duration and lacked detailed neuropathy phenotyping^20–23^. In this study we compare the change in CCM and IENFD with symptoms, signs, QST, autonomic function and neurophysiology over 6.5 years in a cohort of patients with diabetes.

## Results

### Clinical and metabolic assessment (Tables 1, 2)

**Table 1:** Clinical and neuropathy parameters in control subjects and patients at baseline.

Variable	Controls (n = 19)	Patients (Baseline) (n = 19)	*p* value
**Clinical and laboratory parameters**			
Age (years)	47.4 ± 14.2	52.5 ± 14.7	0.20
Duration of diabetes (years)	NA	26.0 ± 13.8	NA
Weight (kg)	80.7 ± 18.0	82.0 ± 19.8	0.9
BMI (kg/m^2^)	27.5 ± 4.0	29.0 ± 5.7	0.50
BP (mmHg)	131 ± 23/74.0 ± 11.0	132 ± 21/ 71 ± 8	0.90/0.50
HbA1c (mmol/mol)	37.5 ± 3	63.5 ± 18.7	**0.0002**
Triglycerides (mmol/l)	1.4 ± 0.7	1.8 ± 1.7	0.9
LDL—C (mmol/l)	2.7 ± 0.9	2.23 ± 0.9	**0.05**
eGFR (ml min/ [1.73 m]^2^)	83 ± 7	82 ± 20	0.70
ACR (mg/mmol)	0.3 ± 0.1	7.5 ± 15.7	**< 0.0001**
**Clinical neuropathy and QST measures**			
NSP (/38)	0.15 ± 0.5	3.5 ± 4.5	**0.0005**
NDS (/10)	0.57 ± 1.0	3.7 ± 2.4	**< 0.0001**
VPT (V)	7.5 ± 6.9	13.0 ± 8.0	0.06
CPT (°C)	28.4 ± 2.3	26.5 ± 3.5	0.51
WPT (°C)	36.9 ± 2.2	40.0 ± 3.7	0.20
CIP (°C)	9.0 ± 8.3	8.0 ± 8.5	0.90
WIP (°C)	45.0 ± 2.8	47.0 ± 2.5	0.15
**Autonomic neuropathy measures**			
DB-HRV (beats/min)	30 ± 12	21 ± 15	**0.005**
Neuropad (%)	91.0 ± 21	62.4 ± 34	0.13
**Nerve conduction studies**			
SNAP (µV)	17.9 ± 9.7	11.41 ± 10.9	0.06
SNCV (m/s)	49.8 ± 4.5	43.5 ± 9.0	**0.01**
PNAP (mV)	6.0 ± 2.2	3.8 ± 1.9	**0.004**
PMNCV (m/s)	48.7 ± 4.1	43.5 ± 3.6	**0.0007**
**Corneal confocal microscopy**			
CNFD (no./mm^2^)	37.7 ± 6.5	28.8 ± 6.5	**< 0.0001**
CNBD (no./mm^2^)	96.5 ± 38.6	67.6 ± 30.2	**0.009**
CNFL (mm/mm^2^)	27.2 ± 3.4	22.2 ± 4.9	**0.0007**
**Skin biopsy**			
IENFD (no./mm)	9.8 ± 3.8	6.6 ± 4.3	**0.04**

BMI—body mass index, BP—blood pressure, HbA1c—Glycosylated haemoglobin, eGFR—estimated glomerular filtration rate, ACR—albumin creatinine ratio, LDL-C—low density lipoprotein cholesterol, NSP—neuropathy symptom profile, NDS—neuropathy disability score, VPT—vibration perception threshold, DBHRV—deep breathing heart rate variability, sural nerve action potential (SNAP), Sural nerve conduction velocity (SNCV), Peroneal nerve amplitude (PNAP), Peroneal motor nerve conduction velocity (PMNCV), CNFD—corneal nerve fibre density, CNBD—corneal nerve branch density, CNFL—corneal nerve fibre length, IENFD—intraepidermal nerve fibre density. Data is presented as mean ± standard deviation (SD). Bold values show statistically significant results. Continuous variables were compared between controls and baseline patient visits using the paired t-test for normally distributed data and Wilcoxon matched-pairs signed rank test for non-normally distributed data.

**Table 2:** Clinical and neuropathy parameters in patients at baseline and follow up.

Variable	Patients (Baseline) (n = 19)	Patients (Follow up) (n = 19)	*p* value
**Clinical and laboratory parameters**			
Age (years)	52.5 ± 14.7	59.5 ± 15.6	NA
Duration of diabetes (years)	26.0 ± 13.8	32.5 ± 13.8	NA
Weight (kg)	82.0 ± 19.8	81.75 ± 18	0.49
BMI (kg/m^2^)	29.0 ± 5.7	28.7 ± 5.2	0.53
BP (mmHg)	132 ± 21/ 71 ± 8	127 ± 20 / 67 ± 9	0.37/0.08
HbA1c (mmol/mol)	63.5 ± 18.7	55.9 ± 12	**0.04**
Triglycerides (mmol/l)	1.8 ± 1.7	1.5 ± 1.1	0.9
LDL-C (mmol/l)	2.2 ± 0.9	1.9 ± 1.2	**0.0009**
eGFR (ml min^−1^ [1.73 m]^−2^)	82 ± 20	69 ± 21	**0.004**
ACR (mg/mmol)	7.5 ± 15.7	41.3 ± 123.6	**< 0.0001**
**Clinical neuropathy measures and QST**			
NSP (/38)	3.5 ± 4.5	5.5 ± 5.7	**0.03**
NDS (/10)	3.7 ± 2.4	4.7 ± 2.5	**0.04**
VPT (V)	13.0 ± 8.0	18.0 ± 9.0	**0.02**
CPT (°C)	26.5 ± 3.5	21.8 ± 9.2	**0.006**
WPT (°C)	40.0 ± 3.7	41. 2 ± 4.8	0.38
CIP (°C)	8.0 ± 8.5	8.0 ± 7.7	0.81
WIP (°C)	47.0 ± 2.5	47.1 ± 2.8	0.622
**Autonomic neuropathy measures**			
DB-HRV (beats/min)	21.0 ± 15.0	19.0 ± 7.0	0.67
LFa/RFa	2.8 ± 2.5	2.7 ± 2.7	0.42
E/I ratio	1.3 ± 0.2	1.2 ± 0.2	**0.004**
Valsalva ratio	1.6 ± 0.7	1.4 ± 0.5	**0.001**
30:15 ratio	1.3 ± 0.1	1.1± 0.1	**0.0003**
Neuropad (%)	62.4 ± 34.0	75.0 ± 31.0	0.47
**Nerve conduction studies**			
SNAP (µV)	11.4 ± 10.9	10.5 ± 11.3	0.75
SNCV (m/s)	43.5 ± 9.0	40.4 ± 7.4	**0.02**
PNAP (mV)	3.8 ± 1.9	3.5 ± 1.9	0.299
PMNCV (m/s)	43.5 ± 3.6	42.4 ± 4.3	**0.03**
**Corneal confocal microscopy**			
CNFD (no./mm^2^)	28.8 ± 6.5	25.6 ± 5.2	**0.03**
CNBD (no./mm^2^)	67.6 ± 30.2	43.7 ± 19.0	**< 0.0001**
CNFL (mm/mm^2^)	22.2 ± 4.9	16.1 ± 3.6	**< 0.0001**
**Skin biopsy**			
IENFD (no./mm)	6.6 ± 4.3	5.2 ± 3.7	**0.04**

BMI—body mass index, BP—blood pressure, HbA1c—glycosylated haemoglobin, e GFR—estimated glomerular filtration rate, ACR—albumin creatinine ratio, LDL-C—low density lipoprotein cholesterol, NSP—neuropathy symptom profile, NDS—neuropathy disability score, VPT—vibration perception threshold, CPT—cold perception threshold, WPT—warm perception threshold, CIP—cold induced pain, WIP—warmth induced pain, DBHRV—deep breathing heart rate variability, LFa/RFa ratio—low frequency area (sympathetic) and respiratory frequency area (parasympathetic) ratio, E/I—expiration/inspiration ratio, Sural nerve action potential (SNAP), Sural nerve conduction velocity (SNCV), Peroneal nerve amplitude (PNAP), Peroneal motor nerve conduction velocity (PMNCV), CNFD—corneal nerve fibre density, CNBD—corneal nerve branch density, CNFL—corneal nerve fibre length, IENFD—intraepidermal nerve fibre density. Data is presented as mean ± standard deviation (SD). Bold values show statistically significant results. Continuous variables were compared between baseline and follow up visits using the paired t-test for normally distributed data and Wilcoxon matched-pairs signed rank test for non-normally distributed data.

Age (*p* = 0.2), weight (*p* = 0.9) and body mass index (BMI) (*p* = 0.5) did not differ significantly between patients and controls and also between patients at baseline and follow up. Systolic (*p* = 0.9, *p* = 0.37) and diastolic (*p* = 0.5, *p* = 0.08) blood pressure did not differ between controls and patients at baseline and between patients at baseline and follow up, respectively. HbA1c was significantly higher in patients with diabetes compared to controls at baseline (*p* = 0.0002) and decreased significantly in patients at follow up (*p* = 0.04). Low density lipoprotein cholesterol (LDL-C) was significantly lower in diabetic patients compared to controls at baseline (*p* = 0.05) and decreased further at follow up (*p* = 0.0009), whilst triglycerides did not differ between patients and controls at baseline (*p* = 0.9) and did not change at follow up (*p* = 0.9). eGFR did not differ significantly between diabetic patients and controls at baseline and decreased at follow up (*p* = 0.004). Albumin creatinine ratio (ACR) was significantly higher in diabetic patients compared to controls at baseline (*p* < 0.0001) and increased further at follow up (*p* < 0.0001).

### Neuropathy assessments

#### Neuropathic symptoms and deficits (Tables 1, 2, Fig. 3)

Neuropathy symptom profile (NSP) (*p* = 0.0005) and neuropathy disability score (NDS) (*p* < 0.0001) were significantly higher in patients at baseline compared to controls and increased significantly (*p* = 0.03, *p* = 0.04, respectively) in patients at follow up.

#### Quantitative sensory testing (QST) (Tables 1, 2*, *Fig. 3)

Vibration perception threshold (VPT), cold perception threshold (CPT), warm perception threshold (WPT), cold induced pain (CIP), warm induced pain (WIP) and percentage colour change in Neuropad did not differ significantly (*p* > 0.05) in patients at baseline compared to controls. Whilst VPT increased (*p* = 0.02) and CPT (*p* = 0.006) decreased significantly there was no change in WPT, CIP, WIP and Neuropad.

#### Electrophysiology (Tables 1, 2, Fig. 3)

Sural (*p* = 0.01) and peroneal (*p* = 0.007) nerve conduction velocity and peroneal nerve amplitude (*p* = 0.004) were significantly lower in patients at baseline compared to controls. Sural (*p* = 0.02) and peroneal (*p* = 0.03) nerve conduction velocity decreased significantly, with no change in sural (*p* = 0.75) or peroneal (*p* = 0.29) nerve amplitudes in patients at follow up.

#### Autonomic neuropathy (Tables 1, 2, Fig. 3)

Deep breathing heart rate variability (DB-HRV) was significantly lower in patients at baseline compared to controls (*p* = 0.005). Expiration/inspiration (E/I) ratio (*p* = 0.004), Valsalva ratio (*p* = 0.001), and 30:15 ratio (*p* = 0.003) increased significantly with no change in DB-HRV (*p* = 0.67) and sympathetic low frequency area (LFa)/parasympathetic respiratory frequency area (RFa) ratio (*p* = 0.42) at follow up.

#### IENFD (Tables 1, 2, Figs. 1 and 3)

Intraepidermal nerve fibre density (IENFD) was significantly lower in patients at baseline (*p* = 0.04) compared to controls and decreased (*p* = 0.04) in patients at follow up.

**Figure 1:**
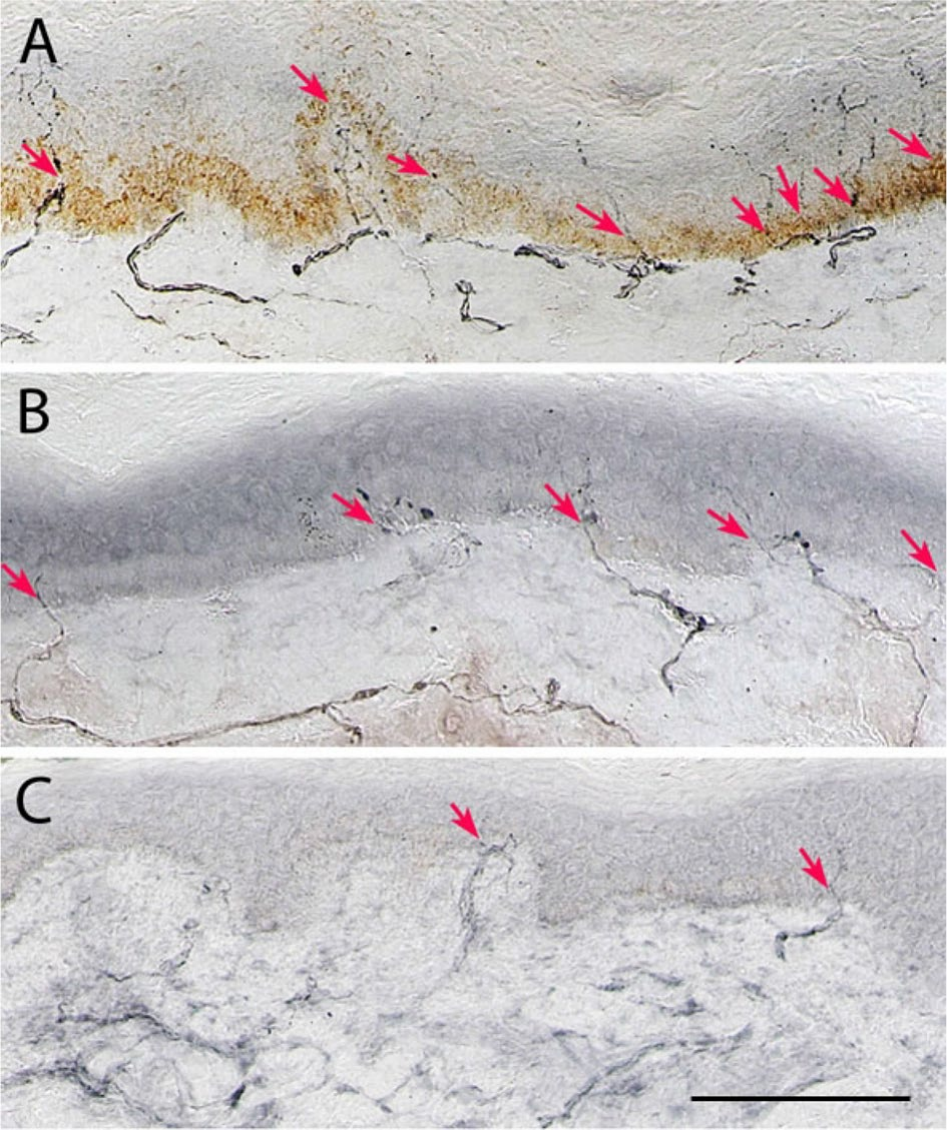
Representative images from skin biopsies from healthy control (**A**) and diabetes patient of similar age at baseline (**B**) and a follow-up visit after 6.5 years (**C**). Note numerous branching nerves reaching top layers of epidermis (**A**; red arrows) and sparse short single nerve and two dividing nerves (red arrows) in epidermis of the baseline biopsy (**B**) and more difficult to discern shorter nerves in the follow-up biopsy (red arrows). Scale bar for A–C = 100 µm.

#### CCM (Tables 1, 2, Figs. 2 and 3)

Corneal nerve fibre density (CNFD) (*p* < 0.0001), Corneal nerve branch density (CNBD) (*p* = 0.009) and Corneal nerve fibre length (CNFL) (*p* = 0.0007) were significantly lower in patients at baseline compared to controls and CNFD (*p* = 0.03), CNBD (*p* < 0.0001) and CNFL (*p* < 0.0001) decreased at follow up.

**Figure 2:**
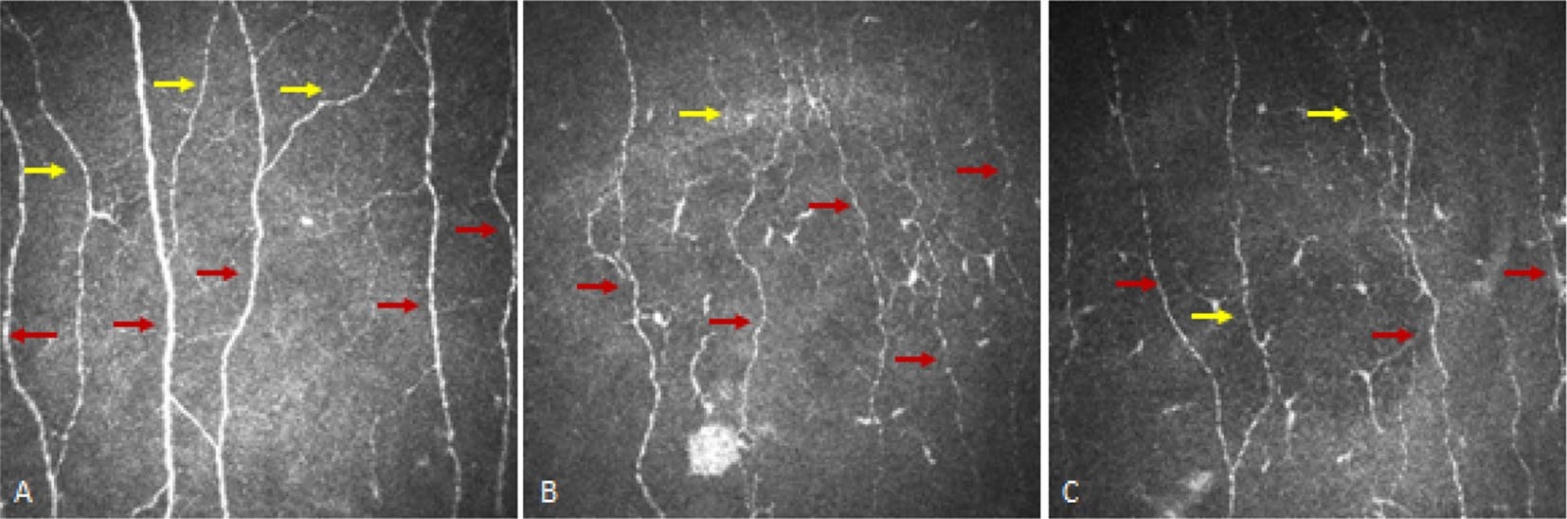
Corneal confocal microscopy image from a healthy control (**A**) and patient with diabetes at baseline (**B**) and follow-up (**C**) showing a progressive loss of nerve fibres (red arrows main nerves, yellow arrows branches) in patients with diabetes.

**Figure 3:**
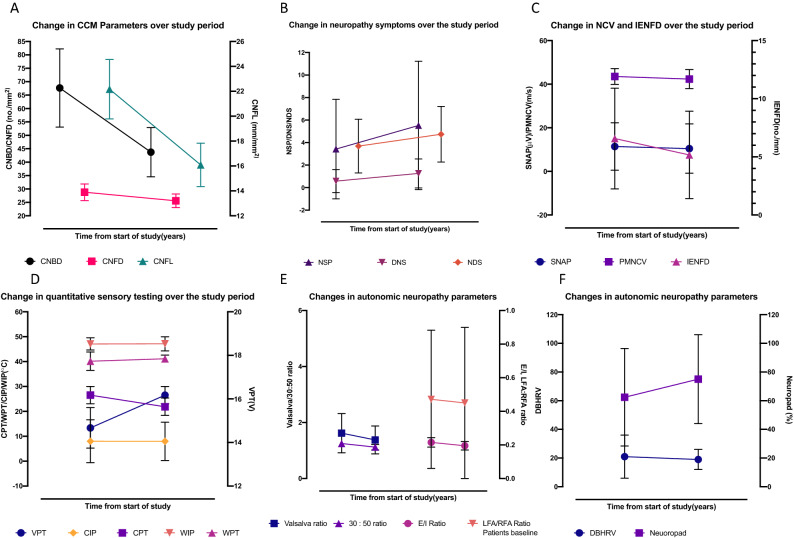
Percentage change from baseline values in CCM parameters (**A**), neuropathy symptoms (**B**), NCV and IENFD (**C**), quantitative sensory testing (**D**) and autonomic neuropathy (**E**,**F**).

#### Associations between the change in clinical and neuropathy measures (Table 3, Fig. 3)

**Table 3:** Correlations between percentage change in small fibre pathology and other measures of diabetic neuropathy from baseline to follow up.

Variable	CNBD	CNFD	CNFL	IENFD
IENFD	**r = 0.53** ***p = 0.02***	**r = 0.62** ***p = 0.005***	**r = 0.56** ***p = 0.01***	
NSP	r = − 0.26 *p* = 0.29	r = − 0.43 *p* = 0.08	r = − 0.045 *p* = 0.86	r = − 0.07 *p* = 0.76
NDS	r = − 0.13 *p* = 0.58	r = − 0.43 *p* = 0.08	r = − 0.11 *p* = 0.66	r = − 0.05 *p* = 0.82
CPT	r = 0.076 *p* = 0.77	r = 0.29 *p* = 0.26	**r = 0.66** ***p = 0.006***	r = 0.27 *p* = 0.26
VPT	**r = − 0.55** ***p = 0.02***	**r = − 0.54** ***p = 0.03***	r = − 0.08 *p* = 0.76	r = − 0.12 *p* = 0.37
DB-HRV	r = − 0.19 *p* = 0.42	**r = − 0.55** ***p = 0.02***	r = − 0.14 *p* = 0.57	r = − 0.03 *p* = 0.87
LFA/RFA ratio	r = 0.26 *p* = 0.27	r = 0.09 *p* = 0.70	r = 0.017 *p* = 0.95	r = 0.13 *p* = 0.58
E/I ratio	r = 0.24 *p* = 0.32	r = 0.31 *p* = 0.21	**r = 0.68** ***p= 0.002***	**r = 0.595** ***p= 0.007***
Valsalva ratio	r = 0.41 *p* = 0.08	r = 0.14 *p* = 0.56	r = 0.25 *p* = 0. 32	**r = 0.59** ***p= 0.008***

NSP—neuropathy symptom profile, NDS—neuropathy disability score, DNS—diabetic neuropathy symptom score, VPT—vibration perception threshold, CPT—cold perception threshold, DB-HRV—deep breathing heart rate variability, LFA/RFA ratio—low frequency area (sympathetic) and high frequency area (parasympathetic) ratio, E/I—expiration/inspiration ratio, CNFD—corneal nerve fibre density, CNBD—corneal nerve branch density, CNFL—corneal nerve fibre length, IENFD—intraepidermal nerve fibre density. Bold values show statistically significant results.

ΔIENFD correlated with age (r =  − 0.56, *p* = 0.01), BMI (r =  − 0.47, *p* = 0.04), waist to hip ratio (r =  − 0.66, *p* = 0.001), ΔE/I ratio (r = 0.595, *p* = 0.0071) and ΔValsalva ratio (r = 0.59, *p* = 0.0078). ΔCNFD correlated with ΔVPT (r =  − 0.54, *p* = 0.03), ΔDBHRV (r = 0.55, *p* = 0.02) and ΔIENFD (r = 0.62, *p* = 0.005). ΔCNFL correlated with ΔCPT (r = 0.66, *p* = 0.006), ΔE/I ratio (r = 0.68, *p* = 0.002) and ΔIENFD (r = 0.56, *p* = 0.014). ΔCNBD correlated with ΔVPT (r =  − 0.55, *p* = 0.02) and ΔIENFD (r = 0.53, *p* = 0.02). There was no correlation between change in HbA1c, lipids and neurophysiological parameters with change in CCM or IENFD (Supplementary Table 1).

## Discussion

In this study we show a progressive worsening of diabetic neuropathy in diabetic patients despite an improvement in HbA1c and LDL cholesterol, although there was no correlation between change in HbA1c, and LDL cholesterol with change in any measure of neuropathy. In T1DM the DCCT showed that intensive glycaemic control reduced the incidence of DPN^3^. However, in patients with T2DM, the UKPDS^4^, VA-CSDM trial^5^ and ACCORD^7^ trials showed no effect of improved glycaemic control on DPN. A major problem in these clinical trials was the end points utilised to assess neuropathy including symptoms and signs of neuropathy and quantitative sensory testing, which were unable to accurately measure change in neuropathy^2^.

Neurophysiology is considered to be the gold standard for the diagnosis of DPN and has been adopted as an endpoint in multiple clinical trials^9^, but has failed to show a significant change in these trials^24^. Indeed, our longitudinal data now shows a relatively small magnitude of reduction in peroneal and sural nerve conduction with no change in amplitudes over 6.5 years. It is therefore not surprising that most trials lasting 12–24 months show no change in neurophysiology.

Small fibre damage usually precedes large fibre damage and contributes to clinically meaningful end-points like painful diabetic neuropathy and foot ulceration due to altered skin blood flow and delayed wound healing^2^. Skin biopsy with IENFD quantification is the current gold standard for the evaluation of small fibre damage^9^ and whilst it is reliable and reproducible it is invasive and resource-intensive^11^. CCM is a rapid, non-invasive and reproducible ophthalmic imaging technique which can be used to objectively quantify small fibre damage in a range of peripheral neuropathies^15,25–29^. We have previously shown comparable diagnostic utility of CCM and IENFD in diabetic neuropathy^19^. Furthermore, in longitudinal studies reduced corneal nerve fibre length predicts incident DPN^30,31^ and those at risk of developing DPN^32^. Indeed, CCM has shown corneal nerve regeneration 6 months after pancreas and kidney transplantation in T1DM with no change in quantitative sensory testing and an improvement in neuropathic symptoms and nerve conduction only after 24 and 36 months, respectively^21,33^. A recent study from Japan showed that an improvement in glycaemic control, body weight and blood pressure in patients with T2DM was associated with an improvement in corneal nerve fibres, neurophysiology and vibration perception over 4 years and correlated with a reduction in HbA1c^34^.

Studies have also shown an association between CCM and LDIflare in healthy control subjects^35^ and with LDIflare, cooling detection thresholds and HRV in patients with diabetes^16^. In the present study CCM measures worsened with greater magnitude than IENFD and large fibre (VPT, CPT, sural and peroneal nerve conduction velocities) and autonomic (E/I ratio, Valsalva ratio and 30:15 ratio) measures of neuropathy. The worsening of corneal nerve fibre measures was associated with worsening of other small fibre measures including cold perception threshold, IENFD and autonomic neuropathy, but not neurophysiology. Indeed, a number of studies have shown corneal nerve loss in patients with diabetic autonomic neuropathy^36–38^ and a correlation between CCM and a wide range of other measures of neuropathy including peroneal and sural nerve conduction^36^ and both cold and warm perception thresholds^16,39^.

A limitation of this study is the relatively small number of patients assessed at follow up. However, the main strength of this study is the comprehensive phenotyping of diabetic neuropathy over 6.5 years, enabling a detailed comparison of the change in small and large fibre measures of diabetic neuropathy.

In conclusion, CCM identifies progressive nerve damage despite an improvement in glycaemic control and LDL cholesterol. Furthermore, corneal nerve loss was associated with a loss of IENFD and worsening of other measures of small fibre neuropathy. CCM is a rapid, non-invasive test to identify progression of neuropathy and may have greater utility than symptoms, signs, QST and nerve conduction studies in longitudinal follow-up studies and clinical trials of DPN.

## Methods

### Participant selection

Nineteen patients with diabetes [type 1 DM (n = 15) and type 2 DM (n = 4)], from the Manchester University Hospital Diabetes Centre and 19 age-matched healthy control participants were recruited and assessed between 2009 and 2011 and at follow up in 2017. The control group comprised of healthy volunteers without DM and were not on any regular medications for any co-morbidities. Patients with a history of neuropathy from any other cause, ocular disease, corneal trauma or surgery, systemic disorders affecting the skin or cornea were excluded. All the tests performed at baseline were repeated in the follow up study using the same protocol and equipment. This study has approval from the Health Research Authority (HRA), North West—Greater Manchester South Research Ethics Committee. Written informed consent was obtained from all individuals prior to participation. This research adhered to the tenets of the declaration of Helsinki.

### Anthropometric and laboratory measurements

All participants underwent assessment of height, weight and body mass index (BMI). Glycated haemoglobin (HbA1c), total cholesterol, low-density lipoprotein (LDL)-cholesterol, triglycerides (TG), serum creatinine and urinary albumin creatinine ratio (ACR) were measured using routine laboratory methods in the Department of Biochemistry, Manchester University NHS Foundation Trust. Estimated glomerular filtration rate (eGFR) was calculated using the abbreviated Modification of Diet in Renal Disease (MDRD) equation: 186 × (creatinine/88.4) − 1.154 × (age) − 0.203 × (0.742 in females) × (1.210 if Afro-Caribbean race).

### Assessment of neuropathy

The neuropathy symptom profile (NSP) was used to assess the symptoms of neuropathy. The modified neuropathy disability score (NDS) which is comprised of an assessment of vibration perception, pinprick, temperature sensation and presence or absence of ankle reflexes was used to evaluate neurological deficits. A Horwell Neurothesiometer (Scientific Laboratory Supplies, Wilford, Nottingham, UK) was used to establish the Vibration Perception Threshold (VPT). Cold (CT) and warm (WT) perception thresholds and cold (CIP) and warm induced pain (WIP) thresholds were tested on the dorsolateral aspect of left foot using the TSA-II NeuroSensory Analyser (Medoc, Ramat-Yishai, Israel). Electrodiagnostic nerve conduction studies (NCS) were undertaken using a Dantec Keypoint System (Dantec Dynamics, Bristol, UK), equipped with a DISA temperature regulator to keep the limb temperature constant at 32–35 °C. The ANX 3.0 autonomic nervous system monitoring device (ANSAR Medical Technologies, Philadelphia, PA, USA) was used to assess deep breathing heart rate variability (DB-HRV), sympathovagal balance via the sympathetic low frequency area (LFa)/parasympathetic respiratory frequency area (RFa) ratio, expiratory/inspiratory (E/I ratio), Valsalva ratio and 30:15 ratio. Sudomotor dysfunction was assessed by quantifying the percentage colour change after applying the Neuropad to the area over the base of the first metatarsal head using our previously established protocol and automated quantification^40^.

### Skin biopsy

Local anaesthetic (1% lignocaine) was applied to the dorsum of the foot, 2 cm above the second metatarsal head and two 3 mm punch biopsies were performed. Sections of 50 µm were stained using anti-human PGP 9.5 antibody (Abcam, Cambridge, UK). SG chromogen (Vector Laboratories, Peterborough, UK) was used to demonstrate nerve fibres and IENFD was quantified using previously established criteria and expressed as the number per millimetre length of epidermis^41^. The follow-up skin biopsy was taken from the same foot, in close proximity to the first biopsy. IENFD was quantified by the same investigator in a masked fashion.

### Corneal confocal microscopy (CCM)

CCM examination (Heidelberg Retinal Tomography III Rostock Cornea Module; Heidelberg Engineering, Heidelberg, Germany) was performed using our previously established protocol^42^. Six non-overlapping images, three per eye, were selected from the centre of the cornea. Three corneal nerve parameters were quantified: Corneal nerve fibre density (CNFD): the total number of major nerve fibres per square millimetre of corneal tissue, corneal nerve fibre branch density (CNBD): the number of branches emanating from the major nerve trunks per square millimetre of corneal tissue and corneal nerve fibre length (CNFL): the total length of all nerve fibres and branches (millimetre per square millimetre) using manual quantification software [CCMetrics (Manchester, UK)]^43^.

### Statistical analyses

Statistical analyses were performed using GraphPad Prism for Mac OS X (version 8.3.0, GraphPad Software, San Diego, California USA, www.graphpad.com). Data were tested for normality using the Shapiro–Wilk normality test. All data are expressed as mean ± standard deviation (SD). Continuous variables were compared between baseline and follow up visits using the paired t-test for normally distributed data and Wilcoxon matched-pairs signed rank test for non-normally distributed data. Ordinary one-way ANOVA was performed (Kruskal–Wallis test was used for non-normally distributed data) to compare between group differences of controls and baseline patient values. Post-hoc corrections for multiple comparison testing was done using Tukey’s test. Correlations were performed between the percentage change in IENFD and CCM parameters and other variables using Pearson’s or Spearman’s Rank Test according to the distribution of the data. A two-way p-value of less than 0.05 was considered to be statistically significant.

## Supplementary Information

Supplementary Information.

## Data Availability

The datasets generated and analysed during the current study are available from the corresponding author on reasonable request.
