# Supplementary material for: Corneal confocal microscopy identifies small fibre damage and progression of diabetic neuropathy
Source: Sci Rep. 2021 Jan 21;11:1859. doi: 10.1038/s41598-021-81302-8 (PMC7820596; doi:10.1038/s41598-021-81302-8)
Supplement: Supplementary file 1 — Supplementary Information. [file 41598_2021_81302_MOESM1_ESM.doc]

**Corneal Confocal Microscopy Identifies Small Fibre Damage and Progression of Diabetic Neuropathy**

Shaishav Dhage^1,2,3^, Maryam Ferdousi^2^, Safwaan Adam^1,2,3^ , Jan Hoong Ho^1,2^, Alise Kalteniece^2^, Shazli Azmi^1,2^, Uazman Alam^4^, Georgios Ponirakis^5^, Ioannis Petropoulos^5^, Andrew J Atkinson^2^, Andrew Marshall^6^, Maria Jeziorska^2^, Handrean Soran^1,2^ and Rayaz A Malik^2,5 *^

^1^Department of Medicine, Manchester University NHS Foundation Trust, Manchester, United Kingdom

^2^Cardiovascular Research Group, University of Manchester, Manchester, United Kingdom

^3^The Christie NHS foundation trust, Manchester, United Kingdom

^4^Institute of ageing and chronic disease, University of Liverpool, Liverpool, United Kingdom

^5^Department of Medicine, Weill Cornell Medicine-Qatar, Doha, Qatar

^6^Department of Clinical Neurophysiology, Manchester University NHS Foundation Trust, Manchester, United Kingdom

***Corresponding author**:

Rayaz A Malik, MBChB, PhD

Professor of Medicine,

Weill Cornell Medicine-Qatar,

Qatar Foundation,

Education City,

Doha, Qatar.

[ram2045@qatar-med.cornell.edu](mailto:ram2045@qatar-med.cornell.edu)

**Supplementary Table 1. Correlations between percentage change in CCM and IENFD and clinical and metabolic variables and neurophysiology between baseline and follow up.**

| **Variable** | **CNBD** | **CNFD** | **CNFL** | **IENFD** |
| --- | --- | --- | --- | --- |
| Age on follow-up (years) | r = - 0.27  p = 0.27 | r = 0.02  p = 0.92 | r = -0.30  p = 0.23 | **r = -0.56**  **p =0.01*** |
| Duration of Diabetes(years) | r = - 0.06  p = 0.79 | r = - 0.33  p = 0.19 | **r = -0.64**  **p = 0.006**** | r = -0.33  p = 0.15 |
| Weight (kg) | r = 0.10  p = 0.67 | r = -0.45  p = 0.06 | r = -0.36  p = 0.15 | r = -0.44  p = 0.05 |
| BMI (kg/m^2^) | r = 0.07  p = 0.76 | r = -0.46  p = 0.06 | r = -0.33  p = 0.18 | **r = -0.47**  **p = 0.04*** |
| Waist to hip ratio(cm/cm) | r = 0.02  p = 0.91 | r = -0.27  p = 0.27 | r = -0.23  p = 0.36 | **r = -0.66**  **p = 0.001**** |
| SBP (mmHg) | r = 0.03  p = 0.89 | r = - 0.07  p = 0.76 | r = -0.35  p = 0.16 | r = -0.05  p = 0.83 |
| HbA1c (mmol/mol) | r = 0.16  p = 0.51 | r = 0.007  p = 0.97 | r = 0.36  p = 0.15 | r = 0.19  p = 0.43 |
| eGFR *(ml min^-1^ [1.73m]^-2^)* | r = 0.26  p = 0.27 | r = 0.36  p = 0.15 | r = 0.33  p =0.18 | r = -0.01  p = 0.94 |
| ACR (mg/mmol) | r = -0.26  p = 0.29 | r = -0.07  p = 0.76 | r = -0.35  p = 0.16 | r = -0.002  p = 0.99 |
| LDL-C (mmol/l) | r = 0.24  p = 0.32 | r = 0.25  p = 0.32 | r = 0.012  p = 0.96 | r = -0.003  p = 0.94 |
| Triglycerides(mmol/l) | r = -0.029  p = 0.90 | r = 0.43  p = 0.08 | r = -0.12  p = 0.64 | r = -0.10  p = 0.66 |
| Peroneal nerve amplitude(m/s) Peroneal nerve amplitude(m/s) | r = 0.08  p = 0.72 | r = 0.25  p = 0.32 | r = 0.34  p = 0.17 | r = 0.12  p = 0.61 |
| Peroneal Nerve Velocity(m/s) | r = - 0.08  p = 0.74 | r = 0.061  p = 0.81 | r = 0.04  p = 0.86 | r = 0.21  p = 0.38 |

BMI-Body mass index, HbA1c- Glycated Haemoglobin, SBP-systolic blood pressure, LDL-C – Low density lipoprotein cholesterol, ACR- Albumin creatinine ratio, eGFR- glomerular filtration rate, CNBD- Corneal Nerve Branch Density, CNFL- Corneal Nerve Fibre Length, IENFD- Intra-epidermal Nerve Fibre Density. Bold values show statistically significant results.
